# Improving Rehabilitation Information-Giving to Intensive Care Unit Survivors to Aid in Physical and Psychological Recovery

**DOI:** 10.7759/cureus.13247

**Published:** 2021-02-09

**Authors:** Armin Fardanesh, Stavroula Stavropoulou-Tatla, Oliver Grassby, Sarah Elliott

**Affiliations:** 1 Medicine, Royal Free Hospital, London, GBR; 2 Surgery, Royal Free Hospital, London, GBR; 3 Medicine, Dorset County Hospital, Dorset, GBR; 4 Physiotherapy, Medway Maritime Hospital, Kent, GBR

**Keywords:** rehabilitation, intensive care unit, post-icu syndrome, nice guidelines

## Abstract

Intensive care unit (ICU) survivors have an increased mortality rate and reduced quality of life associated with post-ICU syndrome: a triad of physical, psychiatric and cognitive decline. Following evidence on the benefits of early rehabilitation, the National Institute of Clinical Excellence (NICE) CG83 guidelines instruct the provision of rehabilitation information to ICU patients before discharge. Only 33% of UK trusts meet these guidelines.

The aim of this project was to reach 100% patient and ICU therapist satisfaction with the rehabilitation information given before ICU discharge at Medway Maritime Hospital, within four months.

Patient and therapist satisfaction was assessed using questionnaires at baseline and following each Plan-Do-Study-Act (PDSA) cycle. In PDSA1, a generalised rehabilitation information booklet was created and distributed to ICU survivors pre-discharge. For PDSA2, a personalised rehabilitation plan completed by therapists was added. During PDSA3, the booklet was enriched with mental health and speech and language therapy sections.

Results showed a shift in patient satisfaction scores, indicating a significant change in the median from 20% at baseline to 87% after PDSA3. This was also reflected in the therapist satisfaction scores, which increased significantly from 60% at baseline to 100%.

The introduction of a generalised information booklet, supplemented with a personalised recovery plan, is an effective way of increasing critical care patient and therapist satisfaction with post-discharge rehabilitation information provision. This should translate to greater patient engagement with rehabilitation and improved long-term outcomes. This is ever more pertinent, as the COVID-19 pandemic will exponentially increase the numbers of ICU survivors at risk of long-term morbidity and mortality.

## Introduction

Problem

The NICE CG83 guideline, titled “Rehabilitation after Critical Illness in Adults”, addresses the prevention of the long-term repercussions of admission to an ICU [1]. Section 1.22 of this guidance instructs the provision of patients with a range of information prior to discharge from ICU, with the intention of supporting patients and their carers with their subsequent recovery. NICE recommends that the information includes advice on physical recovery, diet and activities of daily living, as well as links to local support services.

Despite information being readily available from reputable online sources, such as ICU steps and individual trusts, information-giving prior to ICU discharge is frequently overlooked [2]. Indeed, a national study demonstrated only 33% of trusts to be fully compliant with the guidelines on pre-discharge care, with less than half providing information covering all the domains mentioned in section 1.22 [3].

To complement rehabilitation, the guidelines additionally recommend the delivery of follow-up services at two to three months' post-ICU discharge [1]. However, these are only offered by 27% of UK trusts due to funding and staffing constraints and are often poorly attended [4-5]. Consequently, post-ICU rehabilitation is often self-lead and unsupported.

Currently, the evidence exploring ways to improve self-rehabilitation following discharge from ICU is limited. Therefore, multiple sources, including the NICE, 2017 Quality standard 158, have highlighted “Information on discharge from hospital” as a priority subject for Quality Improvement [4,6].

This project was undertaken in the Medway Maritime Hospital ICU in Kent, South-East England. Prior to the start of this study, patients were given diaries during their stay, following a previous project [7]. This aimed to refresh patients’ memory and improve long-term psychological outcomes. Patients were additionally offered the option of a follow-up appointment with the therapy team, two to three months post-discharge. Nevertheless, the provision of formalised information on post-discharge rehabilitation was not a common practice. Thus, this project aimed to improve the quality and provision of information given to patients leaving the Medway ICU. More specifically, the goal was to increase patient and ICU therapist satisfaction with rehabilitation information to 100% within four months.

Background

On average, patients surviving the ICU have a mortality rate of 30% at one year, which remains increased up to 15 years following discharge. Also reported is a lasting reduction in quality of life, which is proportional to the length of their stay [8-9]. This can be attributed partially to post-ICU syndrome, a triad of long-term physical, psychological, and cognitive decline [10].

Post-ICU syndrome affects up to 70% of survivors [11]. These patients often exhibit generalised muscle weakness, with an associated 4% reduction in muscle mass for each day spent in the unit. Additionally, 60% of patients admitted to the ICU suffer from chronic cognitive decline, akin to mild Alzheimer’s disease [12]. The lasting psychiatric implications of an ICU stay are perhaps the best-documented. In fact, 30% of patients are depressed 12 months post-discharge, with many also developing anxiety and post-traumatic stress disorder (PTSD) [10]. Moreover, malnutrition is commonly reported, with some losing nearly 20% of their baseline body weight during an ICU admission [13].

The overall deficit in functional capacity, often permanent, limits patients’ independence. Indeed, 50% of patients will not return to work within a year [14]. The financial burden extends beyond the patient themselves to the immediate family, 20% of whom give up their job to become informal full-time carers [14].

Early rehabilitation has shown some benefit in combating physical weakness and associated muscle loss in ICU survivors, particularly in the short term [15-16]. Furthermore, the implementation of clinical psychological programmes and ICU diaries can help minimise symptoms of depression, anxiety and PTSD [17]. Whilst these can translate to improved health-related quality of life, there is a lack of strong conclusive evidence to suggest that the implementation of early rehabilitation significantly benefits long-term outcomes [18].

Several studies have also highlighted the benefits of continuous rehabilitation post-ICU discharge, both in the form of supervised programmes and self-lead, home-based interventions. The noted benefits included significantly improved exercise capacity, cognitive function and psychological outcomes, all impaired in post-ICU syndrome [17,19-20].

The need for rehabilitation to be continued throughout recovery post-ICU discharge has long been recognised and is currently recommended by NICE for patients with ongoing needs [1,21].

## Materials and methods

Measurement

The quantitative measures used in this study were determined based on the NICE, 2017 Quality standard 158 on post-ICU rehabilitation. This set of statements outline the quality measures and population inclusion criteria to be used in a quality improvement project in this area [6]. More specifically, the satisfaction level with information on post-discharge rehabilitation of ICU patients and that of ICU rehabilitation therapists was set as the primary and secondary outcome measures, respectively. The ICU rehabilitation team was made up of therapists, including physiotherapists, occupational therapists and dietitians working in critical care. The proportion of ICU therapists providing written rehabilitation information constituted the process measure. The balancing measures considered were the cost of an information booklet and the length of the consultation time by therapists.

Results for the primary outcome measure were collected via telephonic interviews with all patients meeting the inclusion criteria. The interviews were conducted in a standardised way, using a questionnaire and a set script to ensure the comparability of the responses. At the end of the interview, additional comments were encouraged, to elicit any information potentially missed. Patients were asked to rate their satisfaction level with physiotherapy, diet and daily life information received separately. Each item was assessed using a 5-point Likert scale, with 1=extremely unsatisfied, 2=unsatisfied, 3=neutral, 4=satisfied and 5=extremely satisfied. The mean satisfaction score across the physiotherapy, diet and daily life domains was used as the primary outcome.

The secondary outcome measure was assessed via a questionnaire distributed to the ICU therapy team, which consisted of physiotherapists, occupational therapists and dietitians. All ICU therapists were contacted for a response apart from the lead therapists from each field, who participated in the booklet creation and patient recruitment. In a similar way to the patients, a 5-point Likert scale was used to measure the satisfaction level of the therapists, regarding the information they provided in their own domain. Additional free text options and multiple-choice questions aimed to establish what information the patients/therapists were receiving/providing and what their preferences would be regarding the information content and format. The therapists were finally asked to comment on the mean time spent providing rehabilitation information, which acted as a qualitative estimate of the balancing measure.

Data collection occurred during a four-week baseline period and a two-week period following each PDSA cycle. The mean satisfaction score with the information provided, reported by patients was 24%(±8) at baseline, whilst that for therapists was 55%(±21). At the same time, only 25% of ICU therapists reported distributing written rehabilitation information to ICU survivors.

Design

Setting

This project was conducted in the nine-bed ICU of Medway Maritime Hospital, in Kent, England, which serves a catchment area of over 300,000 people.

Aim

This study aimed to significantly increase the satisfaction of ICU survivors and critical care therapists, with regards to the information provided to guide post-discharge rehabilitation. More specifically, the target was to achieve a median satisfaction level of 100% within four months.

Inclusion Criteria

The study population included ICU survivors at risk of morbidity, who were discharged during the four-week baseline period or during the two-week PDSA cycles. Clinical assessments were undertaken by the lead rehabilitation team therapists based on the defining criteria of morbidity risk, set by the aforementioned quality standard by NICE and presented in Table 1 [6].

**Table 1:** The definition of physical and non-physical morbidity risk for ICU patients, as per the NICE Quality standard 158, used as the inclusion criteria for this study ICU: intensive care unit; NICE: National Institute of Clinical Excellence [6]

\ADULTS IN CRITICAL CARE AT RISK OF MORBIDITY	
Physical	- Anticipated long duration of critical care stay. - Obvious significant physical or neurological injury. - Unable to self-ventilate on 35% oxygen or less - Presence of premorbid respiratory or mobility problems. - Risk or presence of malnutrition, changes in eating patterns. - Poor or excessive appetite, inability to eat or drink. - Unable to get in and out of bed independently. - Unable to mobilise independently over short distances.
Non-Physical	- Recurrent nightmares, particularly where patients report trying to stay awake to avoid nightmares. - Intrusive memories of traumatic events that have occurred before admission (for example, road traffic accidents) or during their critical care stay (for example, delusion experiences or flashbacks). - Acute stress reactions including symptoms of new and recurrent anxiety, panic attacks, fear, low mood, anger or irritability in the crucial care unit. - Hallucinations, delusions and excessive worry or suspiciousness. - Expressing the wish not to talk about their illness or changing the subject quickly to another topic. - Lack of cognitive functioning to continue to exercise independently.

Approach

**Figure 1:**
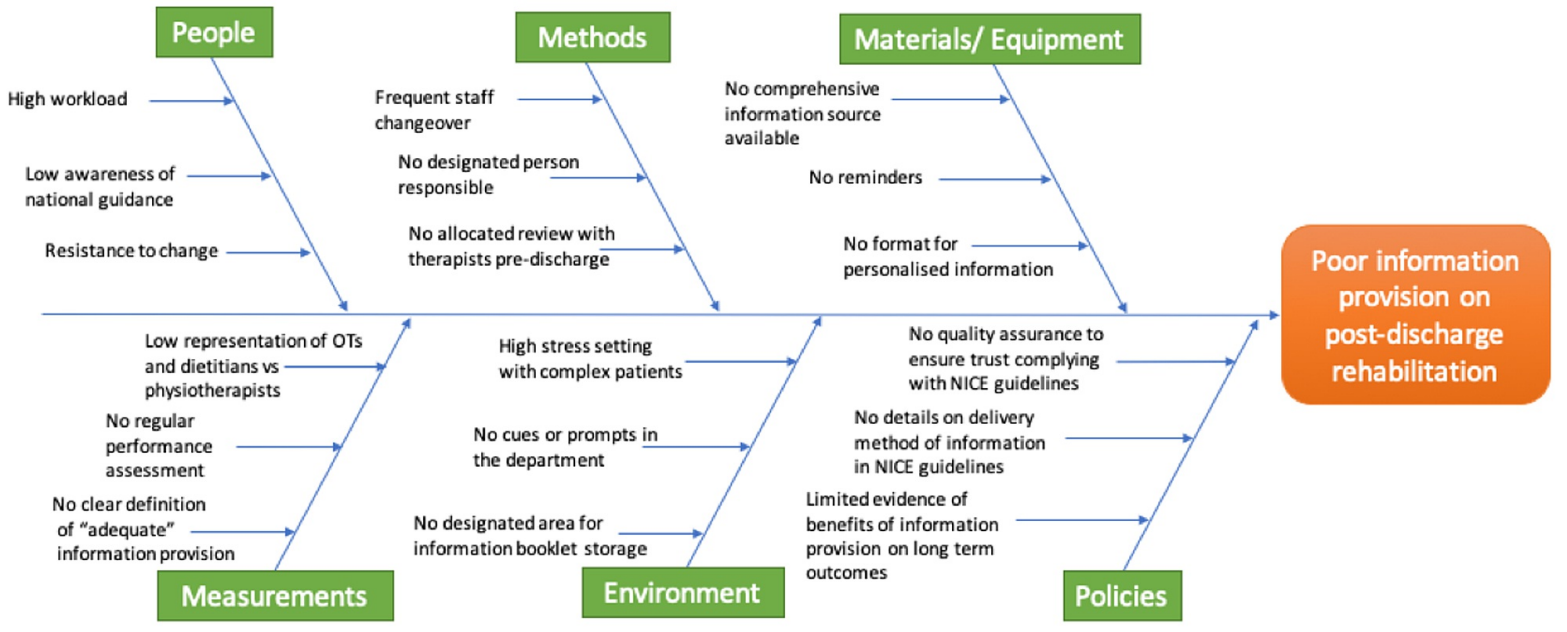
A cause and effect fishbone diagram illustrating the factors underlying poor distribution of rehabilitation information to patients being discharged from ICU The factors underlying poor distribution of rehabilitation information to patients surviving the ICU were determined from focus group meetings with rehabilitations staff and ICU clinicians as well as interviews with patients.

This study used the Plan-Do-Study-Act (PDSA) format, a structure for quality improvement projects recommended by National Health Service (NHS) Improvement. The changes introduced were designed to be sustainable, readily implemented and minimally disruptive to previous ICU practices. A cause-and-effect fishbone diagram was constructed identifying the various barriers to information provision (Figure 1), using data obtained from baseline collection from patients and therapists, along with focus group meetings with the lead rehabilitation team therapists, and intensive care clinicians. This was used to determine the interventions implemented.

The study team included three fourth-year medical students from King’s College London, supervised by a consultant physiotherapist.

Partial Patient and Public Involvement

This study was supported by patients who were contacted at each cycle. Feedback received at baseline and at each cycle was used to assess the effectiveness of each intervention and help design the next one.

Statistical Analysis

All statistical analyses were performed in Sigmaplot 14.0 (Systat Software, San Jose, California) and Microsoft Excel (Microsoft Office 2016, Microsoft Corporation, Redmond, WA). Unless otherwise stated, data are presented as the mean (+/- SD) and compared to the baseline using an unpaired two-way, two-sample t-test (SD= standard deviation).

Strategy

Given the low satisfaction levels with information provision at baseline and the reported lack of written information, in PDSA1 (29/11/18 - 12/12/18), an information booklet titled “Going Home after Critical Care” was introduced (extracts of which are represented in the figures in the Appendices). This booklet was created with the guidance of the lead therapists of the ICU rehabilitation team. The booklet offered insight into the recovery process and provided exercise, diet and daily life recommendations in line with the NICE CG83 guidelines [1]. Included was safety-netting information, links to useful resources and the contact details of the critical care rehabilitation team at Medway Maritime Hospital to schedule a follow-up appointment, where outpatient rehabilitation plans were amended. Despite the marked increase in satisfaction levels observed after PDSA1, several patients described sections of the booklet not to be applicable to them due to various underlying health conditions.

**Figure 2:**
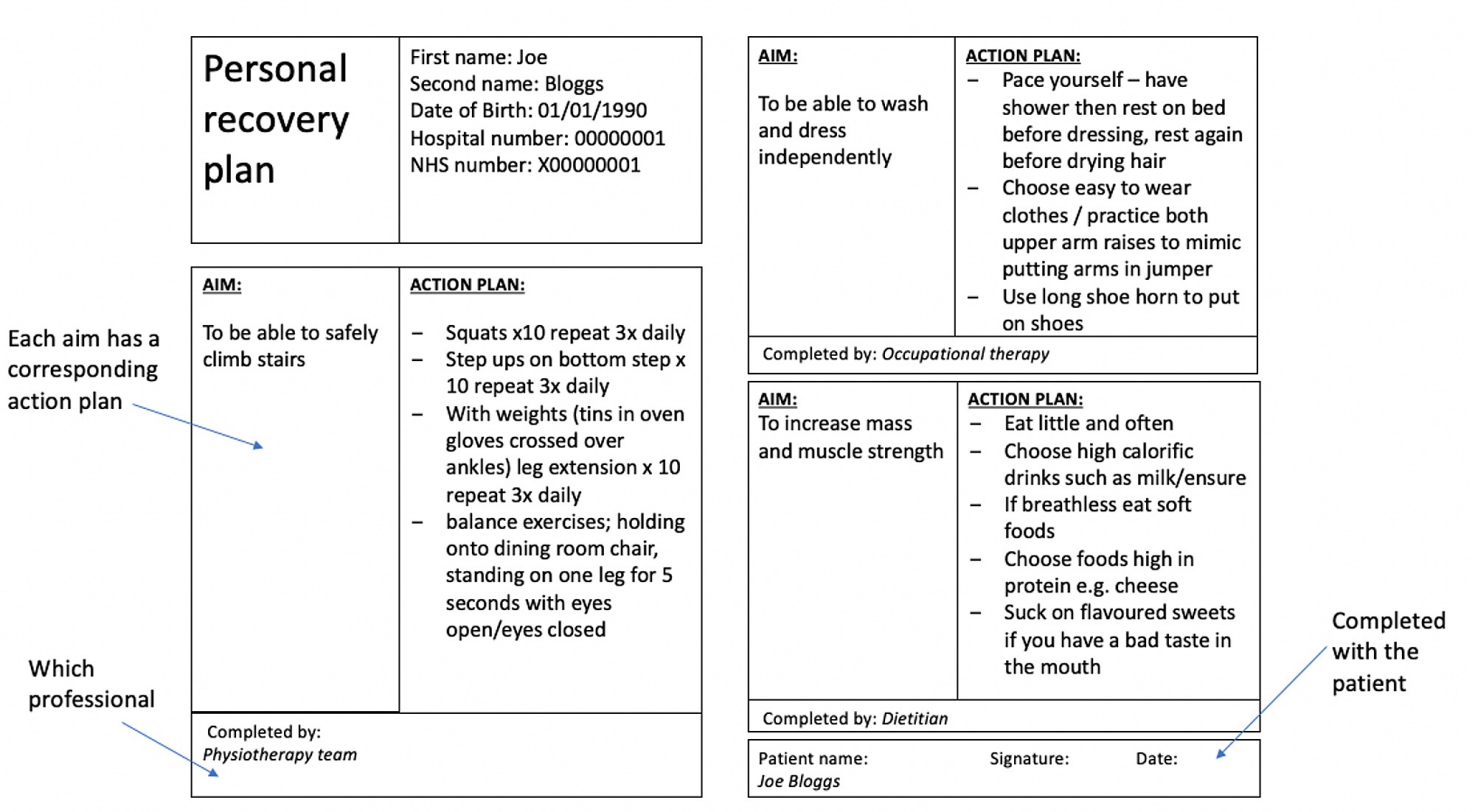
Sample personalised recovery plan completed for a typical patient Each aim has a corresponding action plan, that can be prescribed by a said therapist. The recovery plans are individualised with the aims specific for that patient's needs (patient details are fictional).

To address this, in PDSA2 (3/1/19 - 16/1/19), a personalised section was added to the booklet. This was in the form of a template for rehabilitation goals and action plans to be completed by the therapists, alongside the patient and their carer (Figure 2). Even though this intervention was well-received by patients and therapists alike, a theme noted in the feedback was the lack of mental health input.

Therefore, in PDSA3 (February 1, 2019 - February 14, 2019), the booklet content was enriched with the help of the rehabilitation team counsellor, as well as the lead speech and language therapist, who overviewed the addition of sections on mental health and recovery from invasive ventilation. The safety-netting and resource links were updated accordingly.

## Results

During the active PDSA cycles (dates detailed above), a total of 21 patients met the inclusion criteria. Out of those, 18 were included in the study, as two were unreachable and one was deceased. Responses were obtained from a total of 15 rehabilitation team therapists.

**Figure 3:**
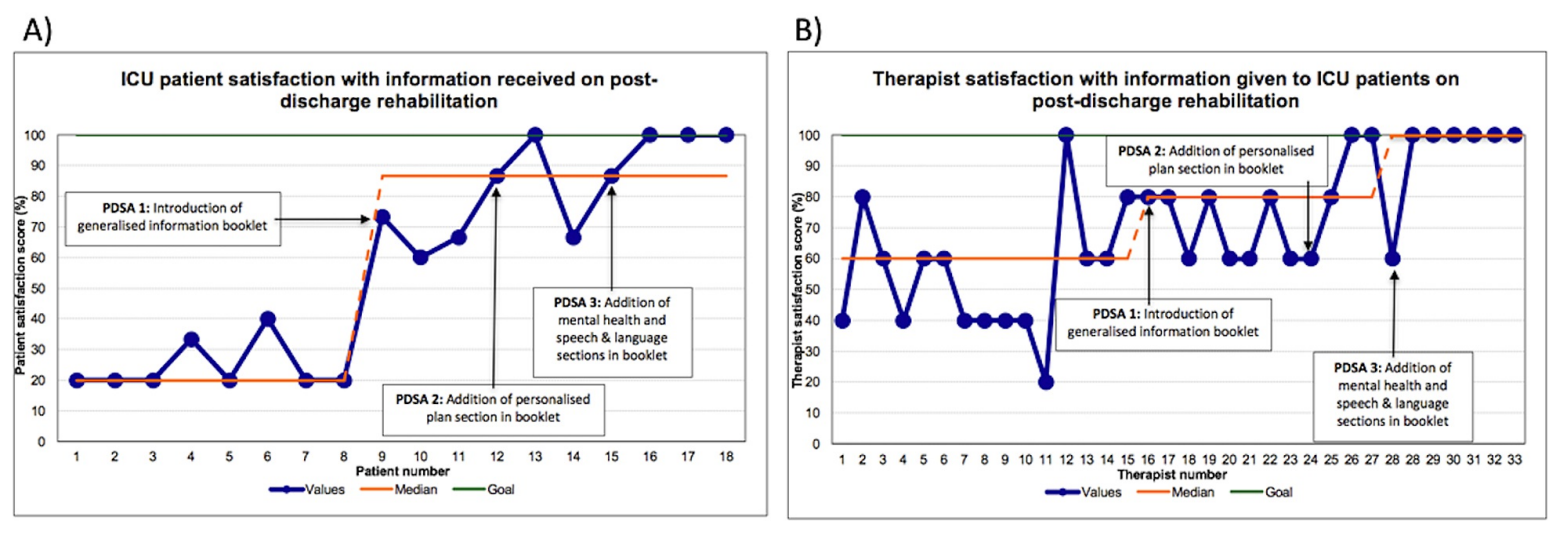
A+B: run chart for the primary outcome measure (A) and secondary outcome measure (B), plotted per patient/therapist participating in the study The blue line indicates the values obtained while the green one demonstrates the outcome goal of 100%. The orange line represents the median. In A, the median was which was replotted from 20% at baseline to 87%, after a shift was observed with 10 consecutive points above the baseline median. In B, the median was replotted from 60% at baseline to 80% and finally 100%, after two shifts were observed with seven and six consecutive points above the previous median. The textboxes describe the changes implemented per PDSA cycle. ICU: intensive care unit; PDSA: Plan-Do-Study-Act

The results of the primary outcome measure are displayed as a run chart in Figure 3A. This illustrates all 10 patient satisfaction scores since PDSA1 being above the baseline median of 20%. Any sequence of six or more consecutive points above/below the median constitutes a shift, which indicates a significant change and mandates for the median to be replotted, in this case at 87% [22]. The target satisfaction of 100% was reached in four cases but was not consistently maintained. Figure 3B illustrates the results of the secondary outcome measure. These showed a shift during PDSA1 and PDSA 2, with seven therapist satisfaction scores exceeding the baseline median of 60%. This indicates a significant increase and allows for the median to be replotted at 80%. During PDSA3, another significant change occurred with six points exceeding the 80% mark, raising the new median to 100% [22]. The target satisfaction of 100% was achieved in eight cases, with six of those in PDSA3.

**Figure 4:**
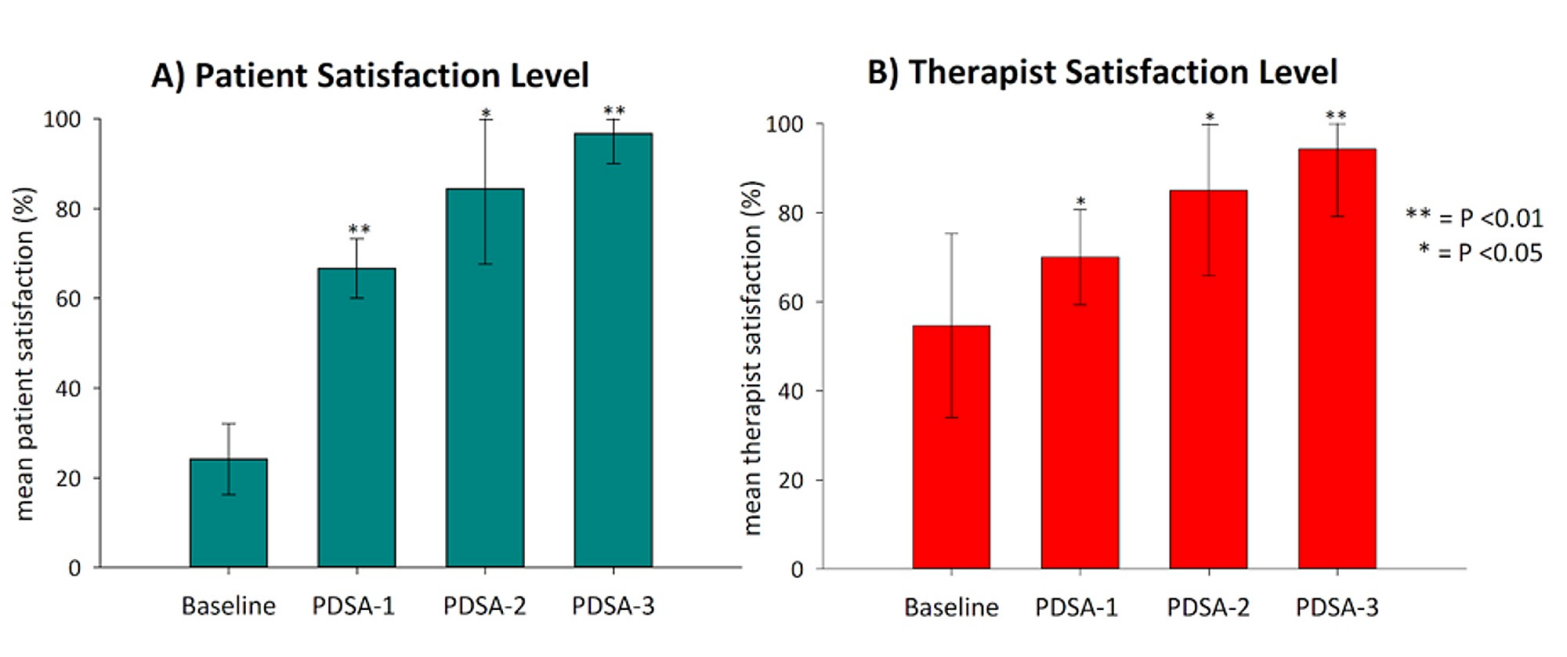
Bar chart illustrating the mean values of the: A) primary and B) secondary outcome measures at baseline and during each PDSA cycle Error bars were used to demonstrate the standard deviation. P-values were calculated with an unpaired, two-way, two-sample t-test comparing each value to the baseline, and represented as * for P<0.05 or ** for P<0.01. PDSA: Plan-Do-Study-Act

As demonstrated in Figure 4A, overall, patient satisfaction scores increased significantly from a baseline value of 24% (±8) to 67% (±7), 84% (±17) and, finally, 97% (±7) after each PDSA cycle, respectively. Similarly, therapist satisfaction scores increased significantly from 55% (±21) at baseline, to 70% (±11) in PDSA1, 85% (±19) in PDSA2 and, finally, 94% (±15) in PDSA3, as exhibited in Figure 4B.

The proportion of ICU therapists providing written rehabilitation information, used as the process measure, increased from 25% at baseline to 75% post PDSA1. Following PDSA2, this increased to 100%, which was maintained throughout PDSA3.

**Figure 5:**
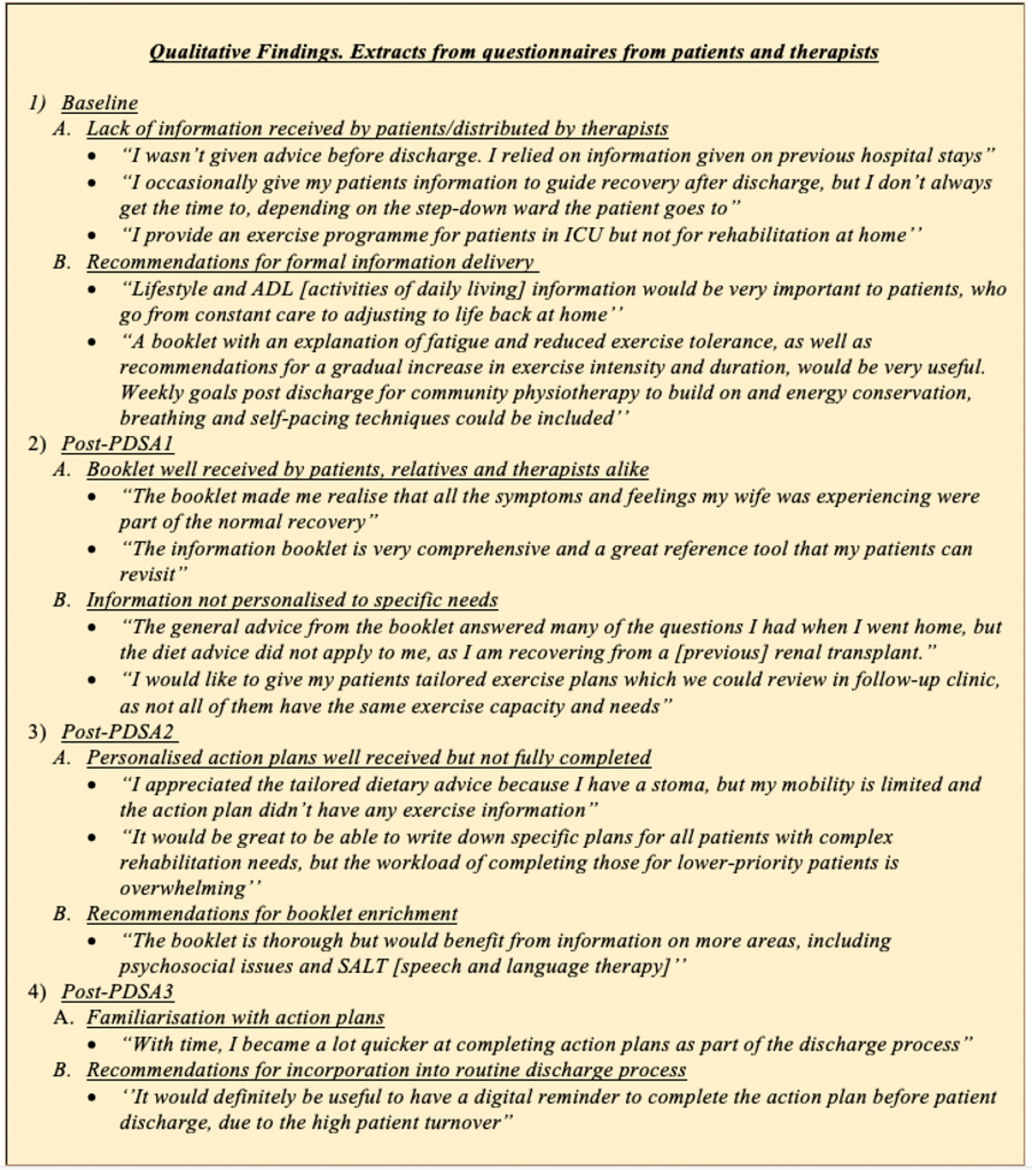
Qualitative results presented as extracts from the patient and therapist questionnaires Analysis of questionnaires demonstrated common themes about the information on post-discharge rehabilitation.

Regarding the mode of information delivery, 44% of patients reported preferring an electronic format, whilst 50% expressed their preference for written information and only 6% opted for verbal delivery. The qualitative data collected from telephone interviews with patients and questionnaires with the therapists are summarised in Figure 5.

In September 2019, the booklet was fully ratified by the Medway Maritime trust board and introduced for continued use, with a provisional review in May 2021. Over the course of 2020, the trust was greatly affected by the pandemic. Due to the unprecedented pressures faced by the ICU department and increased patient turnover, formal measurement of satisfaction did not occur. ICU therapists confirmed that booklets continued to be distributed and were well-received by ICU survivors, of which the majority were recovering from COVID-19.

## Discussion

Lessons and limitations

Explanation of Results

This study involved the creation of a booklet, including expert advice, which successfully addressed section 1.22 of the CG83 NICE guideline, regarding information provision to guide the post-discharge rehabilitation of critical care patients [1]. This was an effective way of increasing both the patient's and therapist's satisfaction with rehabilitation information being distributed to ICU survivors. The addition of a personalised action plan to the booklet further increased the outcome measures. Overall, the close cooperation with the rehabilitation team, devised by the ICU therapists, in conjunction with the enrichment of the booklet content, were decisive factors in the gross improvement in patient and therapist satisfaction rates ultimately observed.

Interestingly, the results showed a discrepancy, most noticeable at baseline, between the satisfaction levels reported by therapists and patients, with the former being higher. The social desirability bias, potentially underlying a hesitancy by the therapists to criticise the quality of their own work could explain this discrepancy. Nevertheless, this bias was most likely minimised, as all the data from the therapists was collected via anonymous questionnaires distributed and collected in a group setting. Therefore, the discrepancy noted is more likely due to other factors, such as the therapists’ expectations being more adjusted to what could be provided given the workload constraints of the ICU setting. An alternative explanation could be poor patient retention of information provided by therapists that was predominantly delivered verbally. The lack of written information is particularly detrimental in the ICU setting, as patients often have considerable memory and concentration deficits.

Impact on Systems

This study introduced a personalised information booklet to structure and support post-discharge rehabilitation, which significantly increased patient satisfaction rates. The improved patient experience is likely to encourage patient engagement with more complete rehabilitation both at home and using outpatient services such as the follow-up clinic. This has the potential to improve patients’ long-term outcomes, resulting in improved quality of life and independence [17,20].

A possible improvement in the long-term outcomes of ICU survivors could also translate to a reduction in the utilisation of NHS resources, which could be of particular economical advantage given the association of ICU stays with higher rates of rehospitalisation, medications, as well as accident and emergency (A&E) and general practitioner (GP) visits [23]. In fact, over 80% of ICU survivors see their GP within six months. Apart from the follow-up of chronic conditions, the GP is the first port of call for common questions about recovery [16]. The booklet serves as a source of information and expert reassurance regarding commonly encountered issues, complementing the role of general practice. Indeed, normalisation of common symptoms is regularly documented as an important factor in supporting patients post-discharge [19,24].

The potential to improve patient outcomes, whilst simultaneously reducing the economic strain on the NHS, is of vital importance, especially at times of crisis such as the current COVID-19 pandemic. There has been an unprecedented influx of patients into critical care, with over 10,000 ICU admissions from September 1 to December 31, 2020. Whilst the loss of life is overwhelming [25], the long-term needs of ICU survivors should not be neglected. Importantly, the average ICU length of stay and need for advanced respiratory support is by far greater for COVID-19 patients, giving rise to increased deconditioning and what has been described as a “tsunami of rehabilitation needs” [8,26]. Hence, the establishment of robust post-discharge rehabilitation programmes has never been more critical [27].

Balancing Measures and Sustainability:

The large-scale printing of the booklet is associated with an increased carbon footprint and an estimated cost of £1 per booklet. These drawbacks could be mitigated through the electronic booklet completion and delivery for the patients who express such a preference. Importantly, this was the case for nearly half the patients. Moreover, from the end of the study date, patients were able to request a copy of the booklet to be emailed to them.

Furthermore, despite their rise in satisfaction, therapists reported finding the additional task of completing personalised action plans for all eligible patients challenging. As demonstrated in an ICU diary study, an increase in workload in the ICU is often met with resistance, as it adds pressure to an already highly stressful role [28]. This was also reflected in the feedback from patients after PDSA2, who reported that the booklet action plan section was not fully completed.

Nevertheless, with time, therapists appeared more engaged with the final intervention, when all patients reported having complete action plans. At the same time, therapists described action plan completion to become another routine task with practice, which was mirrored in their satisfaction that rose during PDSA3. Indeed, the therapist satisfaction gained from seeing patients benefit from an intervention has been recognised as one of the factors driving compliance [28].

Limitations and Future Work

The main limitation of this study was the limited sample size, as the project was held in a district general hospital with a nine-bed unit. However, this was expected given the inclusion criterion of being at increased risk of morbidity, which leads to the non-inclusion of patients with ICU stays too short to confer a high morbidity risk, very long-term admissions that were not discharged during the study period and naturally those who passed away in the hospital or shortly after discharge. Nevertheless, the sample size was comparable to that reported in a previous study in the unit, if the above factors and failure to reach patients are taken into account [7].

Future work should include the formal incorporation of the completed booklet into the ICU discharge process. Inserting a tick-box requirement into the electronic discharge note and holding regular team meetings will act as reminders for action plan completion and booklet provision, whilst aiding the acclimatisation of new team members. As we have demonstrated the benefits locally, before the COVID-19 crisis, it will be insightful to expand the project to more trusts, including tertiary centres, to assess the impact of this study on a larger sample size, including COVID-19 survivors.

Recent systematic reviews have criticised the quality, scarcity and lack of standardisation of existing evidence on the benefits of post-discharge rehabilitation of ICU survivors [29-30]. Whilst this study illustrated the positive impact of a personalised rehabilitation booklet on patient satisfaction and potentially promoted engagement with post-discharge rehabilitation, any long-term health benefits are yet to be confirmed. Further research in this area is warranted, as more conclusive evidence on the long-term benefits of rehabilitation after ICU discharge is likely to increase adherence with the NICE guidelines.

## Conclusions

This project demonstrates that the creation of a generalised information booklet, including a personalised action plan section, is a simple and effective way of significantly increasing both ICU patient and therapist satisfaction with rehabilitation information provision prior to discharge. Thus, it is a successful means of addressing the NICE CG83 guidelines, section 1.22. To ensure the sustainability of the intervention, regular meetings briefing the rehabilitation team can be held, a reminder can be incorporated into the patient electronic discharge notes and the option of electronic booklet distribution can be introduced.

Whilst no firm conclusions can be drawn given the scarcity of high-quality evidence, improved patient satisfaction is likely to promote engagement with rehabilitation long term, which could potentially translate to improved health-related quality of life and reduced consumption of healthcare resources. This is of vital importance, as the current COVID-19 pandemic has led to a surge of ICU survivors with long-lasting, complex, physical and psychological rehabilitation needs, adding to the socio-economic impact of the crisis.

## Figures and Tables

**Figure 6:**
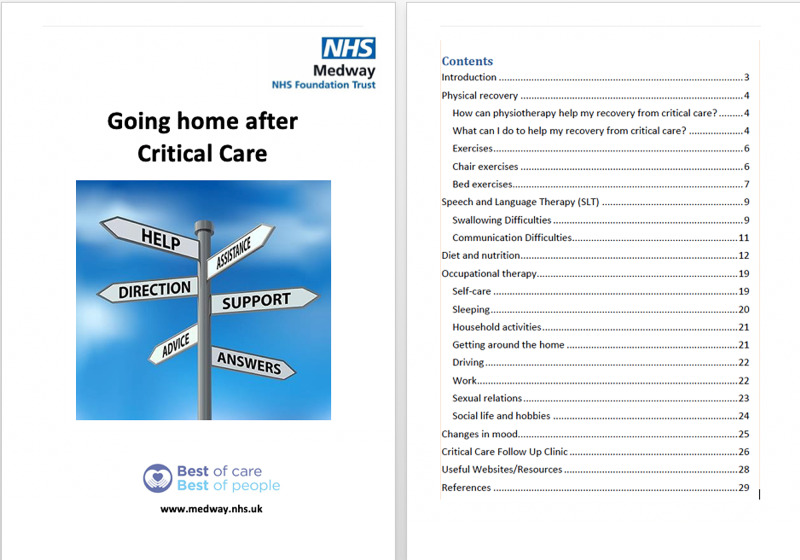
Extract 1 - cover page and contents page of the rehabilitation booklet

**Figure 7:**
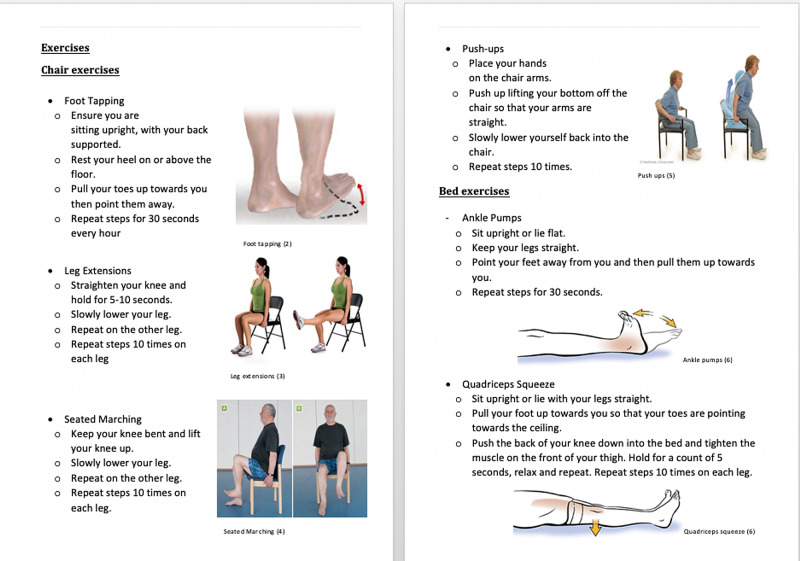
Extract 2 - page 1 of the exercise section in the rehabilitation booklet

**Figure 8:**
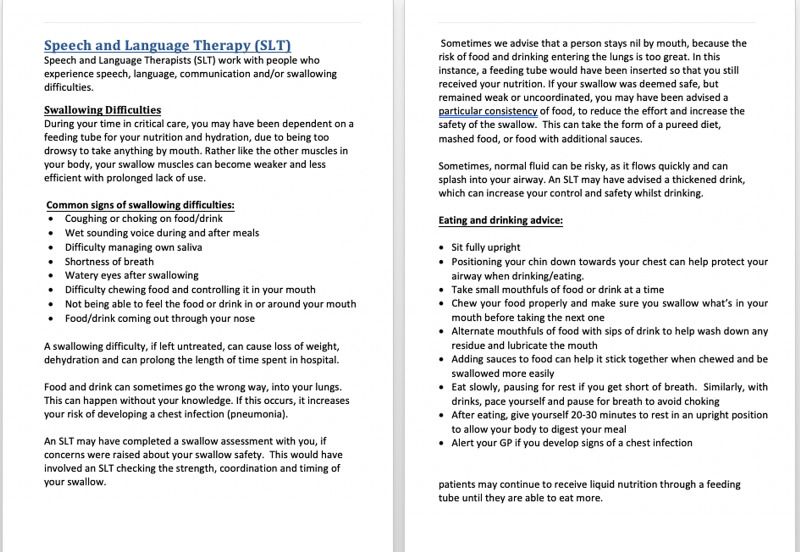
Extract 3 - speech and language therapy and safe swallow section in the rehabilitation booklet

**Figure 9:**
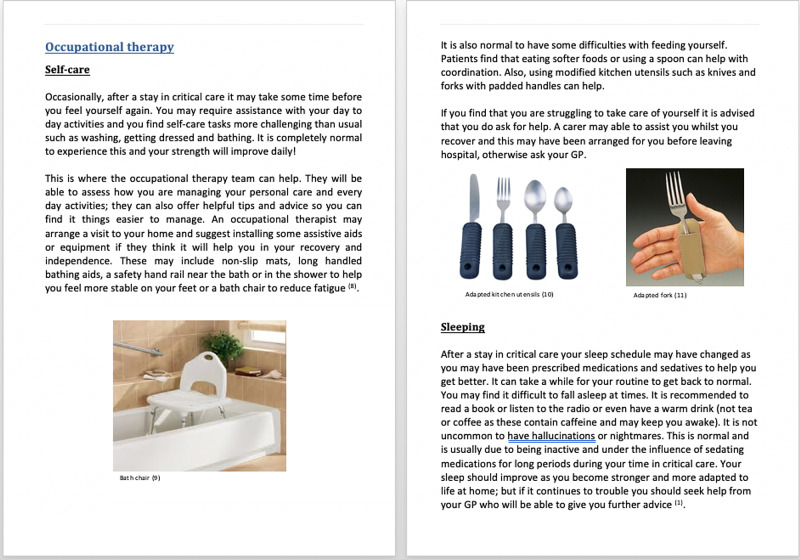
Extract 4 - occupational therapy section in the rehabilitation booklet

**Figure 10:**
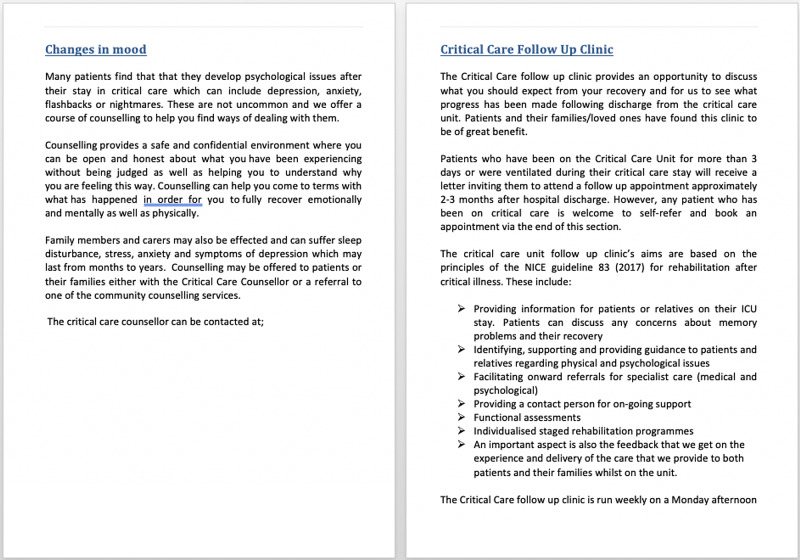
Extract 5 - page on mental health input and follow-up clinic information in the rehabilitation booklet

## References

[1] (2009). NICE. Rehabilitation after critical illness in adults. Clinical guideline (CG83). https://www.nice.org.uk/guidance/cg83.

[2] (2020). ICUsteps. Guide to intensive care. https://icusteps.org/guide.

[3] Berry A, Cutler LR, Himsworth A (2013). National survey of rehabilitation after critical illness. J Intensive Care Soc.

[4] Connolly B, Douiri A, Steier J, Moxham J, Denehy L, Hart N (2014). A UK survey of rehabilitation following critical illness: implementation of NICE clinical guidance 83 (CG83) following hospital discharge. BMJ Open.

[5] Griffiths J, Barber V, Cuthbertson B, Young J (2006). A national survey of intensive care follow‐up clinics. Anaesthesia.

[6] (2017). NICE. National Institute for Health and Care Excellence: Rehabilitation after critical illness in adults. Quality standard (QS158). https://www.nice.org.uk/guidance/qs158/chapter/Quality-statement-3-Information-on-discharge-from-hospital.

[7] Veloso Costa A, Padfield O, Elliott S, Hayden P (2019). Improving patient diary use in intensive care: a quality improvement report. J Intensive Care Soc.

[8] Moitra VK, Guerra C, Linde-Zwirble WT, Wunsch H (2016). Relationship between ICU length of stay and long-term mortality for elderly ICU survivors. Crit Care Med.

[9] Steenbergen S, Rijkenberg S, Adonis T, Kroeze G, van Stijn I, Endeman H (2015). Long-term treated intensive care patients outcomes: the one-year mortality rate, quality of life, health care use and long-term complications as reported by general practitioners. BMC Anesthesiol.

[10] Jackson JC, Pandharipande PP, Girard TD (2014). Depression, post-traumatic stress disorder, and functional disability in survivors of critical illness in the BRAIN-ICU study: a longitudinal cohort study. Lancet Respir Med.

[11] Myers EA, Smith DA, Allen SR, Kaplan LJ (2016). Post-ICU syndrome. Rescuing the undiagnosed. JAAPA.

[12] Wolters AE, Slooter AJ, van der Kooi AW, van Dijk D (2013). Cognitive impairment after intensive care unit admission: a systematic review. Intensive Care Med.

[13] Herridge MS, Cheung AM, Tansey CM (2003). One-year outcomes in survivors of the acute respiratory distress syndrome. N Engl J Med.

[14] Griffiths J, Hatch RA, Bishop J, Morgan K, Jenkinson C, Cuthbertson BH, Brett SJ (2013). An exploration of social and economic outcome and associated health-related quality of life after critical illness in general intensive care unit survivors: a 12-month follow-up study. Crit Care.

[15] Fuke R, Hifumi T, Kondo Y (2018). Early rehabilitation to prevent postintensive care syndrome in patients with critical illness: a systematic review and meta-analysis. BMJ Open.

[16] Schweickert WD, Pohlman MC, Pohlman AS (2009). Early physical and occupational therapy in mechanically ventilated, critically ill patients: a randomised controlled trial. Lancet.

[17] Jones C, Bäckman C, Capuzzo M (2010). Intensive care diaries reduce new onset post traumatic stress disorder following critical illness: a randomised, controlled trial. Crit Care.

[18] Tipping CJ, Harrold M, Holland A, Romero L, Nisbet T, Hodgson CL (2017). The effects of active mobilisation and rehabilitation in ICU on mortality and function: a systematic review. Intensive Care Med.

[19] Jones C, Skirrow P, Griffiths RD (2003). Rehabilitation after critical illness: a randomized, controlled trial. Crit Care Med.

[20] Jackson J, Ely EW, Morey MC (2012). Cognitive and physical rehabilitation of ICU survivors: results of the RETURN randomized, controlled pilot investigation. Crit Care Med.

[21] (1989). Intensive care in the United Kingdom: report from the King's Fund panel. Anaesthesia.

[22] Provost LP, Murray S (2011). The Health Care Data Guide: Learning From Data for Improvement. https://books.google.co.uk/books?hl=en&lr=&id=5JscBgAAQBAJ&oi=fnd&pg=PR25&ots=2gXG8yArV5&sig=x8sYjqybzzR2YZJ5yfmZ2YiV9Kg&redir_esc=y#v=onepage&q&f=false.

[23] Jeitziner MM, Zwakhalen SM, Hantikainen V, Hamers JP (2015). Healthcare resource utilisation by critically ill older patients following an intensive care unit stay. J Clin Nurs.

[24] Prinjha S, Field K, Rowan K (2009). What patients think about ICU follow-up services: a qualitative study. Crit Care.

[25] (2021). ICNARC Report on COVID-19 in critical care. https://www.icnarc.org/Our-Audit/Audits/Cmp/Reports.

[26] Thornton J (2020). Covid-19: how coronavirus will change the face of general practice forever. BMJ.

[27] Stam H, Stucki G, Bickenbach J (2020). Covid-19 and post intensive care syndrome: a call for action. J Rehabil Med.

[28] Johansson M, Wåhlin I, Magnusson L, Hanson E (2019). Nursing staff's experiences of intensive care unit diaries: a qualitative study. Nurs Crit Care.

[29] (2018). 2018 surveillance of Rehabilitation After Critical Illness in Adults (Nice Guideline CG83). Surveillance report.

[30] Connolly B, Salisbury L, O'Neill B (2016). Exercise rehabilitation following intensive care unit discharge for recovery from critical illness: executive summary of a Cochrane Collaboration systematic review. J Cachexia Sarcopenia Muscle.

